# Survivin a radiogenetic promoter for glioblastoma viral gene therapy independently from CArG motifs

**DOI:** 10.1186/s40169-017-0140-y

**Published:** 2017-03-01

**Authors:** George E. Naoum, Zeng B. Zhu, Donald J. Buchsbaum, David T. Curiel, Waleed O. Arafat

**Affiliations:** 1 Alexandria Comprehensive Cancer Center, Alexandria, Egypt; 2 Division of Human Gene Therapy, University of Alabama at Birmingham, Birmingham, AL USA; 3 Department of Radiation Oncology, University of Alabama at Birmingham, Birmingham, AL USA; 4 Cancer Biology Division, Washington University School of Medicine, St. Louis, MO USA; 5 Clinical Oncology Department, Alexandria University, 3 Azarita Street, Alexandria, 21131 Egypt

**Keywords:** Survivin, Promoter, Gene therapy, CArG motifs, Glioblastoma multiforme, Transcription regulation

## Abstract

**Background:**

Radiogenetic therapy is a novel approach in the treatment of cancer, which employs genetic modification to alter the sensitivity of tumor cells to the effect of applied radiation.

**Aim:**

To select a potent radiation inducible promoter in the context of brain tumors and to investigate if CArG radio responsive motifs or other elements in the promoter nucleotide sequences can correlate to its response to radiation.

**Methods:**

To select initial candidates for promoter inducible elements, the levels of mRNA expression of six different promoters were assessed using Quantitative RTPCR in D54 MG cells before and after radiation exposure. Recombinant Ad/reporter genes driven by five different promoters; CMV, VEGF, FLT-1, DR5 and survivin were constructed. Glioma cell lines were infected with different multiplicity of infection of the (promoter) Ad or CMV Ad. Cells were then exposed to a range of radiation (0–12 Gy) at single fraction. Fluorescent microscopy, Luc assay and X-gal staining was used to detect the level of expression of related genes. Different glioma cell lines and normal astrocytes were infected with Ad survivin and exposed to radiation. The promoters were analyzed for presence of CArG radio-responsive motifs and CCAAT box consensus using NCBI blast bioinformatics software.

**Results:**

Radiotherapy increases the expression of gene expression by 1.25–2.5 fold in different promoters other than survivin after 2 h of radiation. RNA analysis was done and has shown an increase in copy number of tenfold for survivin. Most importantly cells treated with RT and Ad Luc driven by survivin promoter showed a fivefold increase in expression after 2 Gy of radiation in comparison to non-irradiated cells. Presence or absence of CArG motifs did not correlate with promoter response to radiation. Survivin with the best response to radiation had the lowest number of CCAAT box.

**Conclusion:**

Survivin is a selective potent radiation inducible promoter for glioblastoma viral gene therapy and this response to radiation could be independent of CArG motifs.

## Background

Brain tumor gliomas are among the most aggressive of human malignancies. Patients with histopathologic subtype, glioblastoma multiform (GBM) have the worst prognosis; despite aggressive surgery, radiation, and chemotherapy [1, 2]. Radiation remains an integral part of the conventional treatment of brain malignancies [3, 4]. However, many gliomas are resistant to radiotherapy [5]. Additionally higher doses of radiotherapy are intolerable to normal brain tissue bringing with it complications that can lead to the deterioration of a patient’s general health. Therefore it is crucial to employ multidisciplinary approaches to overcome such obstacles and offer new solutions on a molecular level.

In this regard, the molecular and genetic basis underlying pathogenesis and treatment resistance of these tumors is under active investigation and is becoming better understood [6]. An important mediator of both tumorigenesis and resistance to treatment involves inhibition of apoptosis [7]. Recently, survivin has been characterized as an important member of the inhibitor of apoptosis family, with a very complex biology that has not been fully understood yet [8]. Survivin expression has been found to be undetectable in normal adult tissues [9]. However, it has been found to be abundantly expressed in a wide variety of human malignancies, including brain tumors [9].

Cancer radiogenetic therapy is a new approach that utilizes a multifunctional platform for tumor imaging, targeting, and gene delivery [10–13]. It employs using genetic modification to alter the sensitivity of malignant or normal tissue to the effect of radiation [14–17]. In this approach many vectors capable of delivering any payload to the tumor cells are designed. Recently, after the success of oncolytic virus in clinical trials and the recent FDA approval for its use in the treatment of melanoma locally [18], it is concluded that viral vectors exhibit great advantages due to their natural capability of efficient cell attachment, entry and high level of transgene expression as part of the viral replication cycle [19–22]. Human Ad-based vectors are now considered a major tool for gene therapy with more than 100 various adenoviral vectors developed for glioma targeting. Additionally, advances in viral based therapy have led to the use of condition replicative adenovirus controlling the replication of the virus mainly through transcription regulation by using tumor specific radioinducible promoters [23, 24].

One of the radiation-inducible promoters described is the RecA promoter that was used to increase tumor necrosis factor-α (TNF-α) production in *Clostridium* sp. [25]. The Egr-1 promoter has also been used as a radioinducible promoter to deliver TNF-α to tumor cells [26–28]. Also, it was studied in the context of radioprotective effect of FLT-3 in severe combined immunodeficient mice [27] for in vitro studies on gene activation [29] and for gene expression in the context of hypoxia inducible promoters [30]. One of the proposed mechanisms of the radiation mediated transcription regulation is the presence of CArG box in the nucleotide sequence of different promoters regions including radiosensitive EGR-1 [31]. Unfortunately, these genes are neither up-regulated in gliomas nor specifically expressed in tumor cells. Nowadays, advances in bioinformatics provide a powerful tool for elucidating the functional features of genes or their promoters, and also prediction tools to identify specific elements within promoters sequence directly with no detectable sequence similarity [32].

To that end, In this study we tried a combined technique of radiation plus transcription regulation to prove the principle of regulating gene expression. The aim of this study is to investigate and select the best radiation inducible promoter in the context of viral brain tumors therapy. We also aim to investigate, using bioinformatics, if the presence of CArG radio responsive motifs or other elements in a promoter’s nucleotide sequences is related to our selection.

## Methods

### Cell culture

The human glioblastoma cell lines D54 MG, U251 MG and human astrocytes (from Dr. Yancey Gillespie, university of Alabama at Birmingham, Birmingham, AL) were maintained in Dulbecco’s modified Eagle’s medium/F12, supplemented with 10% fetal calf serum, l-glutamine (200 μg/mL), 100 U/mL penicillin and 100 μg/mL streptomycin, at 37 °C in a 100% humidified 5% CO_2_ atmosphere.

### Initial screening for mRNA copies in response to radiation

Six different human promoters (FLT-1, VEGF, Cox2, INOS, DR5 and survivin) were assessed for expression of mRNA in D54 MG cells 2 h after exposure to 2 Gy radiation using quantitative RT-PCR. Radiation provided using a ^60^Co therapy unit (Picker, Cleveland, OH).

### Construction of adenovirus with proposed radiation inducible promoter

We constructed recombinant CMV Ad and Ad/Luc (encoding the luciferase reporter gene, a kind gift of Robert D. Gerard university of Leuven, Belgium) Ad/GFP (encoding the reporter green fluorescent protein) or Ad/LacZ (encoding the Escherichia coli b galactosidase gene, provided by De-chu Tang, university of Alabama at Birmingham, Birmingham, AL) driven by different promoters; 0.51 kb of CMV [33], 0.26 kb of survivin [34], 2.6 kb of VEGF promoter region [35], 1.2 kb of DR5 [36], flt-1 as described before [37]. Reporter genes replaced the E1A region in these vectors, under control of human promoters [36, 38]. These replication-deficient adenoviral vectors were constructed based on homologous recombination between pCMV, pVEGF, pDR5, FLt-1 promoter, or pSurvivin shuttle vectors and pVK500 adenoviral backbone that contain the entire adenoviral genome with E1A region deleted from it. Viruses were propagated in 293 cells, purified by centrifugation in CsCl gradients, and plaque titered in 293 cells following standard protocols [39].

### Validation of radiation inducible adenovirus vector and selection of potent radiation inducible promoter in context of adenoviral vector

D54 MG cells were plated per well in 6-well plates at 2 × 10^5^. Then, cells were infected with different multiplicity of infection (MOI) of different (promoter) Ad or CMV Ad then cells were exposed to a range of radiation (0–12 Gy) at single fraction. Control cells were left without radiation. Fluorescent microscopy, luciferase assay, and X-gal staining of the corresponding genes were used to detect the level of expression of related genes to control cells and radiation treated cells after 2 h of radiation and 24 h of radiation to select a potent radiation inducible promoter in context of adenoviral vector.

### Reporter gene assays

#### X-gal assay

Cells were seeded in 24-well plates in quadruplets at a density of 5 × 10^4^ cells/well. The following day, the cells were infected with AdDR5-LacZ at an m.o.i. of 100 in DMEM with 2% FBS for 1 h and then maintained in complete medium. For staining, wells were washed with 1 mM MgCl_2_ in PBS, and cells were fixed by 0.5% (w/v) glutaraldehyde at room temperature, then stained using X-gal reaction solution, and incubated at 37 °C until a color change was obtained. The end absorbance was then measured at 420 nm using a V Max plate reader (Molecular Devices Corp., Sunnyvale, CA, USA). LacZ activities were normalized for differences in incubation times.

#### EGFP expression assay

Cellular EGFP expression was quantitatively examined by FACS analysis and visualized using fluorescent microscopy. Cells were collected 48 h after Ad/VEGF-EGFP infection and approximately 10,000 cells were illuminated at 488 nm and fluorescence was detected in the FITC (525/20 nm) channel. Nonspecific fluorescence was detected using a 575/30 nm emission filter in the PI channel. EGFP fluorescence is expressed as the mean fluorescence signal in EGFP-positive cells in relative units [18] after subtraction of background fluorescence. An Olympus IX70 inverted microscope system (Olympus America, Melville, NY) was used for the screening of EGFP expression in cell monolayers.

### Quantitative reverse transcription-PCR and PCR

Total RNA (5 μg) was isolated by RNeasy kit (Qiagen) and used for cDNA synthesis by oligo [18] and SuperScript II RNase H reverse transcriptase (Invitrogen). Primers used in the reverse transcription-PCR (RT-PCR) assays were described before [40, 41]. Quantitative real-time PCR using SYBR Green PCR Master Mix (Applied Biosystems) was performed with (Bio-Rad) systems according to the manufacturer’s protocol.

### Detection of luciferase expression

Standard single luciferase assay (Promega, Madison, WI) was used to measure firefly luciferase enzyme activity as per the manufacturer’s instruction. For compensation of the differences in cell numbers in different samples, Bradford protein assay (Bio-Rad, Hercules, CA) was used to measure the protein concentration of the lysates. Luciferase is presented as normalized to cellular protein concentration.

### Confirmation of selectivity of survivin as radiation inducible promoter in glioma

In order to detect the optimum dose of radiation for further applications, D54 MG glioma cell lines were plated in six-well plated. The cells were then infected with either AdSurvivin-Luc or AdCMV-Luc (1000 viral particles per cell). Virus-containing medium was replaced with fresh growth medium, after 1-h adsorption. After 24 h, cells were irradiated with different doses of 0, 2, 4, 8, and 12 Gy using a ^60^Co therapy unit (Picker, Cleveland, OH) at a dose of 80 cGy/min. Twenty-four hours later, cells were analyzed by luciferase assay to select the optimum radiation dose. To ensure the effect of this selected dose in different glioma cell lines and to detect the difference of survivin expression levels between tumor and normal cells the same steps were repeated on D54 MG, U251 MG and human astrocytes cell lines. Cells were irradiated with the convention selected dose of 2 GY using a ^60^Co therapy unit (Picker, Cleveland, OH) and analyzed after 24 h by luciferase assay.

All the assays were carried out in quadruplets and several independent experiments were performed to verify the reproducibility of the results.

### Bioinformatics analysis to detect radiation inducible elements

We used the NCBI Nucleotide blast database (https://www.ncbi.nlm.nih.gov/nucleotide/), in order to identify the nucleotide sequence of mRNA transcripts of different available in-house promoters. These included: 1-survivin (NCBI Reference Sequence: NM_001012271.1), 2-FLT-1 (NCBI Reference Sequence: XM_017020485.1), 3-COX-2 (NCBI Reference Sequence: Gene Bank: U20548.1), 4-DR-5(NCBI Reference Sequence: XM_017013944.1), 5-VEGFA (NCBI Reference Sequence: NM_003376.5), 6-iNOS (NCBI Reference Sequence: NM_001204218.1).

The CArG motifs were previously described as motifs which respond to radiation and are responsible for radiation mediated transcription regulation [31]. Also, it was described that CCAAT box (a cis acting element) are significantly present with lower frequencies in radio responsive genes in comparison to regular genes [42].

Therefore we used NCBI FASTA sequence finder to detect the Number of CAAG motifs and the 10-nucleotide motif of consensus sequence in the CArG [CC (A+T rich) 6GG or serum response element] in these promoters. We also screened the promoters using the same software for CCAAT box consensus sequence in attempts to determine a cis acting elements responsible for survivin response to radiation.

### Statistical analysis

Results are expressed as mean ± SD. Student’s *t* test was used according to the distribution of experimental values. *P* values of <0.05 were accepted as significant differences between groups.

## Results

### Evaluation of mRNA accumulation as an indicator of radiation mediated transcription regulation

**Fig. 1:**
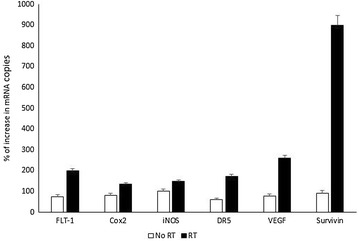
Effect of radiation on mRNA expression of different genes promoters

Six different human promoters (FLT-1, VEGF, Cox2, INOS, DR5 and survivin) were assessed for expression of mRNA in D54 MG cells 2 h after exposure to 2 Gy radiation using quantitative RT-PCR. Levels of expression of FLT-1, DR5, VEGF, INOS and Cox2 increased twofold to threefold, while the survivin gene was upregulated to ~10 folds as depicted in (Fig. 1), indicating a strong radiation-inducible promoter. *Columns,* mean; *P* < 0.05, compared with non-radiated control. However, additional elevation of survivin RNA copy number was reported after 24 h of radiation.

### Radiotherapy increases level of gene expression in the context of adenovirus with radiation relevant promoter in brain tumor

**Fig. 2:**
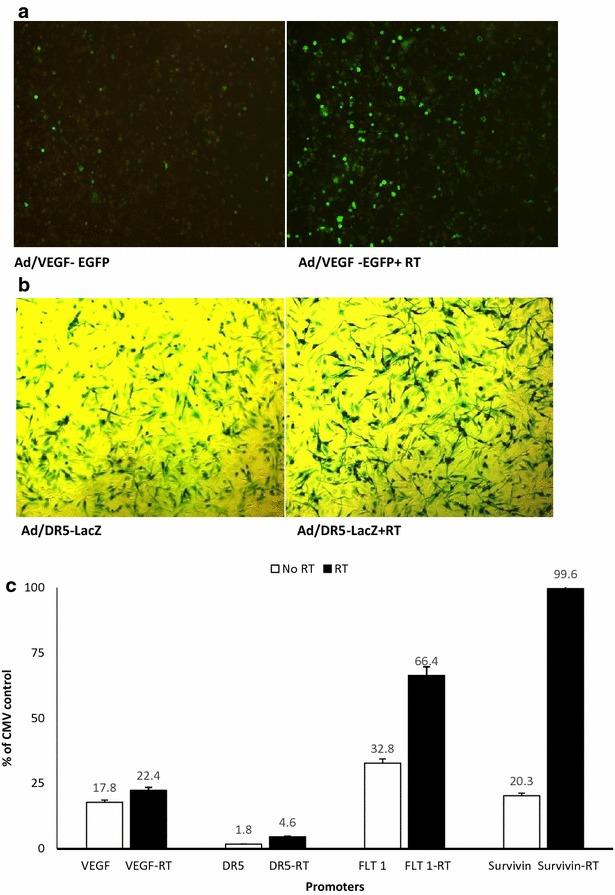
**a** Fluorescent microscopy detecting effect of radiation on GFP reporter gene driven by VEGF promoter. **b** X-gal staining detecting effect of radiation on LacZ reporter gene driven by DR5 promoter. **c** Effect of radiation on different promoters as detected by Luciferase assay

Adenoviral constructs containing VEGF, DR5, FLT-1 and survivin promoter driving lacZ, GFP and luciferase reporter genes were used for infection of D54 MG cells. Fluorescent microscopy was used to detect the effect of radiation on VEGF promoter in Ad/GFP as depicted in (Fig. 2a). X-gal staining used to detect the effect of radiation on DR5 promoter in Ad/LacZ as depicted in (Fig. 2b). Total luciferase activity was measured and values were normalized to amounts of total protein. Experiments were performed in quadruplets using replication-deficient adenovirus. CMV promoters a strong constitutive gene promoter, producing high basal levels of reporter gene expression but shows a negligible increase on irradiation [43]. Therefore, results are shown before and after 24 h of radiation, as a percentage of CMV promoter-induced luciferase expression. We have found that Ad/VEGF-Luc and AdDR5-Luc AdFLT1-LUC showed an increase luciferase expression in response to radiation by 1.25-fold in VEGF, 2.5 fold in DR5 and twofold in FLT-1, as depicted in (Fig. 2c). Ad/survivin-Luc however showed a fivefold increase in response to radiation suggesting also strong radiation-inducible promoter. *Columns,* mean; *P* < 0.05, compared with non-radiated control. For quantification, maximum projections and total fluorescence measurements were performed with Image J and the corrected total cell fluorescence (CTCF) was calculated using the formula CTCF = Integrated Density − (Area of selected cell × Mean fluorescence of background readings) data not shown.

### Radiation induces different levels of gene expression by adenovirus with survivin promoter at different doses

**Fig. 3:**
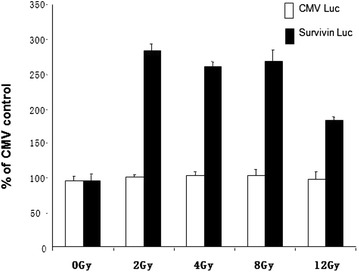
Effect of different doses of radiation on gene expression driven by survivin promoter

To detect the optimal dose of radiation, glioma cell lines (D54 MG) were infected with constructed adenoviral particles (1000 viral particles per cell) containing the *luciferase* gene under either control of survivin or CMV promoters. After viral infection, we conducted a dose–response assay exposing cells to different fractions of radiation as shown in (Fig. 3) and grown for an additional 24 h. Total luciferase activity was measured and values were normalized to amounts of total protein. Experiments were performed in quadruplets. The maximum response to radiation exposure was observed at dose 2 Gy (~3-fold) with higher doses, the response diminished to 1.5-fold, as depicted in Fig. 3.

We therefore considered 2 Gy the optimum dose for radiation. *Columns*, mean; *bars,* SD, *P* < 0.05, compared with non-radiated control.

### Radiation induces high level of gene expression by adenovirus with survivin promoter in different glioma cell lines

**Fig. 4:**
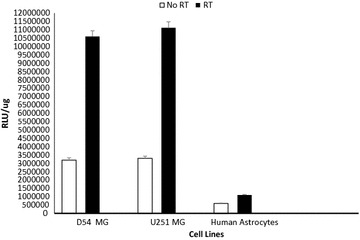
Selectivity of survivin promoter to Glioma cell lines

Different glioma cell lines D54 MG, U251 MG and human astrocytes were infected with Ad-survivin and exposed to the selected dose of 2 Gy. The luciferase activity was assessed before radiation and 24 h after radiation. Survivin promoter showed an increase in expression from 30 to 99% in glioma cell lines after 2 Gy of radiation compared to non-irradiated cells as depicted in (Fig. 4). The difference in survivin expression between glioma cell lines and normal human astrocytes suggest that survivin is a tumor selective promoter. *Columns*, mean; *bars*, SD, *P* < 0.05, compared with non-radiated control.

### CARG Radiation inducible elements and other cis acting elements detection

After using NCBI nucleotide database to identify the nucleotide base sequence of the promoters used in this experiment, FASTA sequence finder was used to determine the presence of elements responding to radiation previously described. Variable number of CAAG elements was detected in the promoters independently from their response to radiation. Also, the CArG box which has been shown to be responsible for egr-1 increased transcription in response to radiation has not been detected in any of the promoters used in this experiment as depicted in Table 1. Therefore we concluded that radiation mediated transcription regulation is not dependent on CArG elements only

**Table 1:** Number of CArG and CAAG elements in different promoters

	Survivin	FLT-1	DR-5	VEGF	COX-2	iNOS
CAAG elements	21	83	58	36	17	167
CArG box [CC (A+T rich) 6GG]	0	0	0	0	0	0

**Fig. 5:**
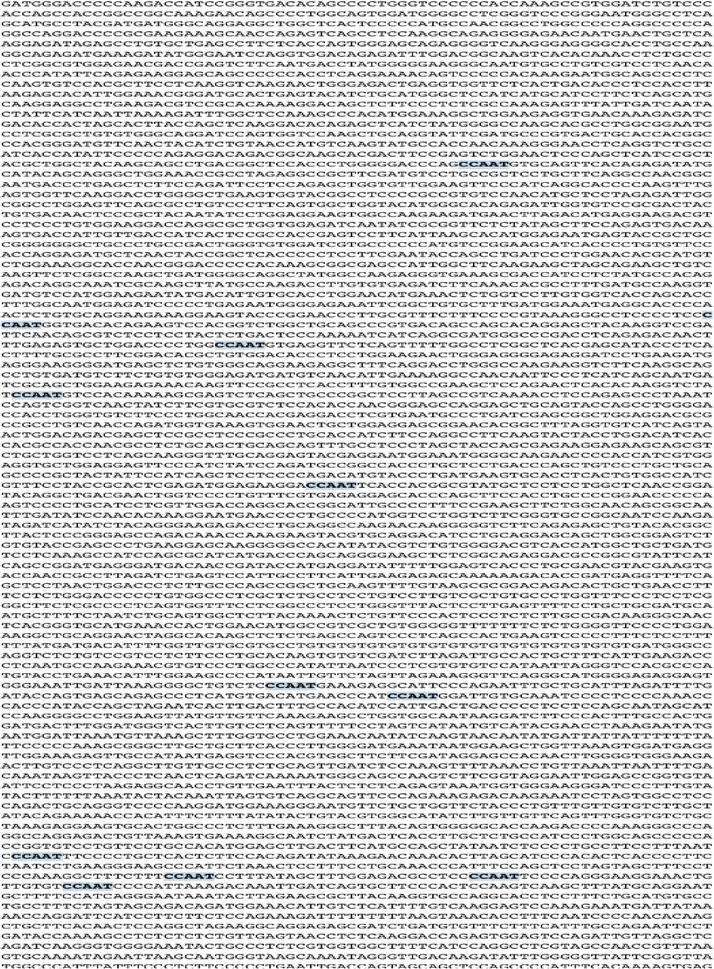
Detetction of CCAAT box (a cis acting element) in iNOS promoter nucleotide sequence

**Fig. 6:**
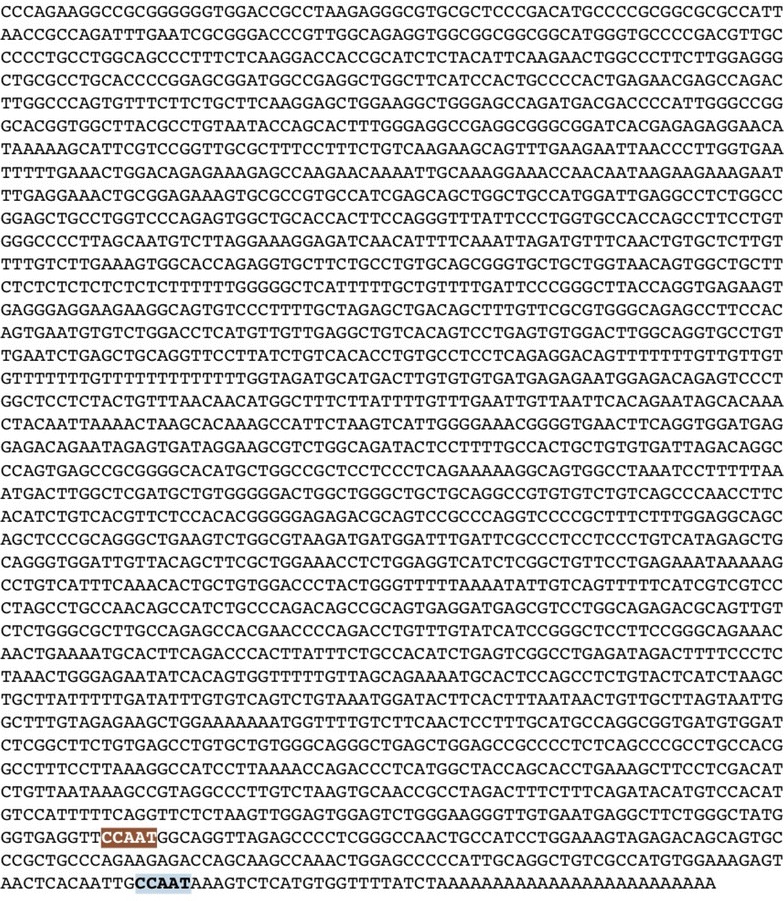
Detetction of CCAAT box (a cis acting element) in Survivin promoter nucleotide sequence

Using NCBI nucleotide database and FASTA sequence finder we screened all the promoters for CCAAT box consensus. iNOS promoter with the lowest response to radiation had higher number of CCAAT (n = 14) as depicted in Fig. 5, while Survivin with the best response to radiation revealed two CCAAT boxes (n = 2) in its sequence as depicted in Fig. 6. The other promoters had numbers of this consensus ranging between these 2 values (data not shown).

## Discussion

Treatment of brain tumors, especially GBM represents a great challenge [44, 45]. The standard GBM treatment includes surgical resection, radiation, and chemotherapy. Although, there has been an emergence of many innovations and advances in chemotherapy and radiation therapy, none of them have prevented recurrence [46, 47]. Rather they have only provided longer survival times. Radiogenetic therapy, is a novel approach that offers a combination model aiming to circumvent the obstacles faced by conventional treatments. It employs targeted viral vectors delivery of therapeutic transgenes under the control of radiation responsive promoters. Enhancing viral replication by using tumor selective promoters is likely to enhance virotherapy [48, 49]. This strategy would restrict therapeutic gene activation to tumor cells and/or irradiated tissues only, thereby providing selective expression for any tumor-located vector. Since gene activation would be controlled, damage to surrounding normal tissue would be reduced, thereby improving the therapeutic ratio replication such as targeting and transcription regulation. In this study we tried a combined technique of radiation plus transcription regulation to prove the principle of regulating gene expression.

Understanding the molecular bases and mechanisms responsible for radiation mediated transcription regulation is crucial for better understanding of this approach and for classifying and selecting the best radioinducible gene promoter. Elements regulating transcriptional process are composed of both *cis*-acting and *trans*-acting elements [50, 51]. The *cis*-acting elements comprise promoters as well as enhancer regions (ex: CArG box, TATA box and CCAAT) that regulate expression of a distinct gene [52]. The *trans*-acting elements are related to a group of transcription factors that bind to specific sites within promoter and/or enhancer regions [53, 54]. Controlling the transcription of therapeutic gene expression [55] by using promoters that are radiation-responsive offers an attractive platform for the combination of radiation therapy and gene therapy [56].

We and others showed in this study, and other studies [57–59], that the survivin promoter in comparison to other promoters may represent an optimal radiation inducible promoter and provide compelling data to suggest that radiation, a mainstay of glioma therapy, further improves survivin-mediated adenoviral gene expression in targeted cells, which is a finding that might have significant implications for patients who may one day be treated with this virus and subsequently receive radiotherapy. Also, the difference in survivin expression levels between glioma cells and normal astrocytes suggest its tumor selectivity and potential safety in sparing normal cells from aggressive therapies.

In attempts to explain the radiosensitivity of survivin promoter, we screened all the promoters used in this experiment for the presence of CArG radio responsive motifs first described in EGR-1 promoter. The egr-1 was the first promoter described more than 2 decades ago to be responsive to radiation [27, 60, 61]. Functional analysis of *Egr*-*1* revealed a number of DNA sequence motifs including GCGGGGGCG that can modulate the radiation-mediated response [31]. Ionizing radiation can readily induce the *Egr*-*1* promoter via a 10-nucleotide motif of consensus sequence in the CArG (CC (A+T rich) 6GG or serum response element) [31, 62–65]. In our study we have analyzed several radio responsive promoters for the CArG elements. None of these sequences were detected in any promoters. Adding to that the number of CAAG sequence in all promoters was independent from response to radiation.

Additionally, our team as well as others have shown that radiation increased the number of survivin RNA transcripts in cells [57, 58, 66]. Therefore we do believe that elements other than CArG may be responsible for survivin radiosensitivity. Of note, it was concluded before that CCAAT box play a gene-specific transcriptional role depending on the type of gene [67]. Since survivin promoter has no TATA box, these sequences are involved in gene expression and cell cycle regulation. Therefore, they might also be responsible for radiosensitivity of survivin. Interestingly, a study by Wu et al. [68] correlated low frequency presence of CCAAT motifs to radio responsive genes. Following this concept we screened the promoters used in these study for this motif consensus. Our results came in accordance to this correlation where survivin promoter, with the best response to radiation, had the lowest number of CCAAT box motifs in comparison to other promoters tested. These preliminary results require future mutational analysis of different promoters in order to explore the mechanism behind radiation mediated transcription regulation and larger sample size to correlate between the different numbers of cis acting element in a given promoter and its response to radiation.

To this end we conclude that survivin is a selective potent radiation inducible promoter for glioma and CArG motifs are not the only elements involved in radiation mediated transcription regulation.
